# Supplementary material for: Biomarkers of Exposure and Potential Harm in Two Weeks of Smoking Abstinence: Changes in Biomarkers of Platelet Function, Oxidative Stress, and Inflammation
Source: Int J Mol Sci. 2023 Mar 27;24(7):6286. doi: 10.3390/ijms24076286 (PMC10093936; doi:10.3390/ijms24076286)
Supplement: Supplementary file 1 [file ijms-24-06286-s001.zip › ijms-2284798-supplementary.pdf]

International Journal of *Molecular Sciences*

MDPI

Supplementary

Biomarkers of Exposure and Potential Harm in Two Weeks of Smoking Abstinence: Changes in Biomarkers of Platelet Function, Oxidative Stress, and Inflammation

Patrudu Makena [1,*], Eric Scott [1], Peter Chen [1], Hsiao-Pin Liu [1,†], Bobbette A. Jones [1,†] and Gaddamanugu L. Prasad [1,2,†]

1 RAI Services Company, 401 N. Main Street, Winston-Salem, NC 27101, USA
2 Prasad Scientific Consulting LLC, Lewisville, NC 27023, USA
* Correspondence: makenap@rjrt.com
† Former employees of RAIS, 401 N. Main Street, Winston-Salem, NC 27101, USA.

Selection of Biomarkers

In addition to the two primary BoPH endpoints leukotriene E_4 (LTE_4) and 2,3-dinor thromboxane B_2 (2,3-d-TXB_2), the Smoking Abstinence Study (SAB) evaluated several additional biomarkers (see Figure 1). These included several established biomarkers of exposure (BoE), which inform of changes in exposure to nicotine and selected harmful and potentially harmful constituents (HPHCs) upon smoking abstinence. 2-cyanoethylmercapturic acid (CEMA), a metabolite of acrylonitrile, was measured as a representative BoE for volatile organic compounds that arise during combustion [1-3]. The biomarkers were measured in 24h urine samples under GLP or other quality conditions, as appropriate. Additionally, complete blood count (CBC) with differential data were collected as part of the safety assessments at baseline and Day 14. A summary of bioanalytical methods is presented in Supplementary Schemes S1, S3 and S4. Only those BoPH that showed consistent and statistically significant changes are discussed in this manuscript.

<u>Additional AA metabolites</u>: It is reported that 11-d thromboxane B_2 (11-dh-TXB_2) is an established BoPH of platelet activation [4]. We also assessed 11-dh-TXB_2 as a part of a panel of arachidonic acid (AA) metabolites, along with the two primary study endpoints. Other AA metabolites, such as 8-isoIP2$F\alpha_{III}$ and PGEM, have been reported to significantly differ between smokers and users of non-combustible tobacco, as well as upon smoking cessation [5-7]. Hence in the SAB study, a panel of AA metabolites consisting of these and the two primary endpoints were assessed.

<u>Arterial blood gases</u>: For the body to function normally, metabolic tissue consumption of oxygen must be equal to the oxygen taken up in the blood through alveolar gas exchange or, metabolic tissue production of CO_2 must be equal to the amount of CO_2 blown off at the alveoli. Chronic cigarette smoking augments the decline in lung function and alters pulmonary ventilation, which leads to impaired gas exchange that is more prominently observed in smokers with COPD. Impaired gas exchange in asymptomatic long-term smokers compared to non-tobacco consumers was reported

previously [8]. Kullmer et al. [9] investigated pulmonary gas exchange during exercise for evaluation of reduced arterial oxygenation at rest in asymptomatic long-term smokers ages 40-60 years. This study demonstrated that even in clinically healthy asymptomatic cigarette smokers a distinct impairment of gas exchange is present, but significant improvement during steady state exercise is also observed. Similar differences in gas exchange between smokers and non-smokers ages 23-69 years were reported by Ouattara et al [10]. However, effect of age on differences in gas exchange in asymptomatic smokers and in short-term smoking abstinence has not been previously demonstrated.

Exhaled Nitric Oxide: Airway inflammation is a common feature of chronic cigarette smoking and invasive procedures may be required for accurate assessment. A noninvasive biomarker, such as fractional exhaled nitric oxide (FeNO), could be a useful biomarker of airway inflammation due to its role in the pathogenesis of several pulmonary diseases including asthma and COPD. Nitric oxide, produced by cells lining the respiratory tract, decreases in the exhaled air of cigarette smokers as an indication of inflammation [11] and FeNO levels increase after smoking cessation [12]. . However, the effect of age on FeNO levels, as investigated in this short-term SAB study, has not been previously described.

Key Inclusion and Exclusion Criteria for the SAB study participants

Inclusion Criteria

- Generally healthy male or female adults, 24 to 60 years of age, inclusive, at the time of consent.
- Exhaled carbon monoxide (ECO) level is $\geq$ 10 ppm and $\leq$ 100 ppm at Screening and Day -2.
- Positive urine cotinine test at Screening and Day -2.
- Smokes only combustible, filtered, non-menthol or menthol cigarettes, 83 mm to 100 mm in length.
- Smokes 10-20 combustible filtered cigarettes per day and inhales the smoke, and has smoked for at least 5 years prior to Screening. Brief periods of abstinence due to illness, quit attempt (30 days prior to Enrollment), or clinical study participation (30 days prior to Enrollment) will be allowed at the discretion of the Investigator. Occasional usage of other tobacco or nicotine containing products may be allowed at the discretion of the Sponsor.
- Willing to abstain from smoking during the study's abstinence period.

Exclusion Criteria

- Presence of clinically significant uncontrolled cardiovascular, pulmonary, renal, hepatic, endocrine, gastrointestinal, psychiatric, hematological, neurological disease, or any other concurrent disease or medical condition that, in the opinion of the Investigator, makes the study participant unsuitable to participate in this clinical study.
- History, presence of, or clinical laboratory test results indicating diabetes.

- Systolic blood pressure of > 160 mmHg or a diastolic blood pressure of > 95 mmHg, measured after being seated for five minutes.

Supplementary Methods

The SAB study evaluated several biomarkers at various timepoints of the study (Figure 1). The urinary BoE consisted of total nicotine equivalents, CEMA, NNAL, NNN, NAT and NAB. Urinary nicotine equivalents is a composite measure of nicotine exposure and was calculated as the molar sum of unconjugated nicotine and its five major metabolites. Plasma nicotine, plasma cotinine and COHb were measured in blood as described previously [13]. The primary endpoints, LTE_4 and 2,3-d-TXB_2, were also measured as a part of a panel of additional AA metabolites.

Arterial blood gases (ABG) and FeNO were measured at baseline and after 14 days of smoking abstinence. Approximately 2 mL of arterial blood was collected from the radial artery of the subjects using a pre-heparinized syringe with needle. Several ABG parameters including PaO_2, $PaCO_2$, O_2 saturation, bicarbonate, and pH were analyzed using a blood gas analyzer (i-STAT System, Abbott Point-of-Care Analyzer) according to manufacturer instructions. FeNO was measured in subjects, as described previously [14], using a point of care device, NIOX VERO®, according to manufacturer instructions.

The CBC with differential counts were performed as part of safety assessments at baseline and 14 days in the SAB study and at the baseline and 8 days in the Vuse study, under CLIA conditions.

Calculation of biomarker changes upon smoking abstinence in the SAB Study: Change of cohort mean (%) from baseline (Day -1) to post-smoking abstinence (Day 7 or Day 14) will be provided for all the biomarkers for both age cohorts. The % change for an age cohort is calculated as:

(Day 7 or Day 14 mean – baseline mean)/ baseline mean x 100

Supplementary Results

SAB Study Conduct

Figure 1 summarizes the study design for the SAB clinical study in which adult smokers abstained from smoking for 14 days in a confinement setting. The SAB was a single-center, two-cohort, smoking abstinence study, in which generally healthy adult male and female smokers participated. Smokers of 10-20 cigarettes per day for at least 5 years prior to screening were recruited. A total of 70 subjects (51 males, 19 females) were enrolled between the two age cohorts (24-34 age cohort, n=33; 35-60 age cohort, n=37). Sixty-eight subjects completed the study and two subjects discontinued early (one for spilling urine during the 24h urine collection and the other for a family emergency). Demographics and characteristics of enrolled subjects are summarized in Table 1. In brief, the mean age of subjects is 30 years in the younger age cohort and 49 years in the older age cohort. The representation of Hispanics was low in both the younger and older age cohorts (9% and 0%, respectively), as was the representation of African Americans (15% and 8%, respectively), reflecting the available subject pool in the area where the study was conducted. Subjects in both age cohorts smoked an average of 17

cigarettes per day, and mean scores on the Fagerström Test for Nicotine Dependence (FTND) were comparable between the younger and older age cohorts (5.5 and 6.0, respectively).

During a 2-day baseline period, subjects smoked their usual brand (UB) cigarettes ad libitum, followed by 14 days of smoking abstinence. No other study products were used in this study. Plasma and urine (24h and spot urine) samples and other biological specimens were collected at baseline and at specified timepoints during the study.

During the 2-day baseline period, which included UB smoking, 39 adverse events (AEs) occurred in 27 subjects (39%). Headache was the most frequently reported AE, experienced by 10 subjects (14%). The Principal Investigator (PI) considered two events of headache in the younger age cohort to be possibly related to study procedures and 37 events not related.

During the abstinence period, a total of 47 AEs were experienced by 27 subjects (39%), including 10 subjects (30%) in the younger age cohort and 17 subjects (46%) in the older age cohort. Back pain was the most frequently reported AE, experienced by five subjects (7%), all in the older age cohort. None of the AEs that occurred during the abstinence period were considered related to study procedures. All AEs that occurred during the study were mild to moderate in severity. No serious AEs or deaths were reported in this study, and no subjects were discontinued due to AEs.

Scheme S1. Bioanalytical Methods for Determining Biomarkers of Exposure.

Constituent	Biomarker	Matrix	Abbreviation	Method	LOQ
**BLOOD**					
**Constituent**	**Biomarker**	**Matrix**	**Abbreviation**	**Method**	**LOQ**
Nicotine[a]	Unconjugated nicotine	Plasma	NIC	LC-MS/MS	0.2 ng/mL
Cotinine[a]	Unconjugated cotinine	Plasma	COT	LC-MS/MS	1.0 ng/mL
Carbon monoxide[b]	Carboxyhemoglobin	Whole Blood	%COHb	Radiometer ABL80 FLEX CO-OX OSM analyzer	0.2%
**URINE**					
**Constituent**	**Biomarker**	**Matrix**	**Abbreviation**	**Method**	**LOQ**
Nicotine[a]	Unconjugated nicotine	Urine	NIC-U	LC-MS/MS	50 ng/mL
	Unconjugated cotinine	Urine	COT-U		50 ng/mL
	Unconjugated trans-3′-hydroxycotinine	Urine	OHCOT-U		50 ng/mL
	Nicotine-N-glucuronide	Urine	NIC-G		50 ng/mL
	Cotinine-N-glucuronide	Urine	COT-G		200 ng/mL
	Trans-3′-hydroxycotinine-O-glucuronide	Urine	OHCOT-G		200 ng/mL
	Total Nicotine Equivalents[c]	Urine	NIC_{Eq}-T	Calculated	N/A
TSNAs[a]					
NNK	4-(N-methylnitrosamino)-1-(3-pyridyl)-1-butanol (NNAL)	Urine	NNAL-T[d]	LC-MS/MS	5 pg/mL
NNN	N-nitrosonornicotine	Urine	NNN-T[d]	LC-MS/MS	0.2 pg/mL
NAT	N-nitrosoanatabine	Urine	NAT-T[d]	LC-MS/MS	5 pg/mL
NAB	N-nitrosoanabasine	Urine	NAB-T[d]	LC-MS/MS	2 pg/mL
CEMA[a]	Acrylonitrile	Urine	CEMA	LC-MS/MS	0.275 ng/mL

[a] Samples were analyzed at Celerion Global Bioanalytical Services, Lincoln, NE.
[b] Samples were analyzed at Celerion Clinical Laboratory Services, Lincoln, NE.
[c] NIC-U and its five metabolites are converted to molar NIC-U equivalents and summed to equal total nicotine equivalents.
[d] The total of unconjugated compound and glucuronide metabolites.
LC-MS/MS - liquid chromatography-tandem mass spectrometry; LOQ - limit of quantitation; TSNAs - Tobacco-specific nitrosamines

Scheme S2. Bioanalytical Methods for Determining the Biomarkers of Potential Harm – Urine.

**URINE**					
**Constituent**	**Biomarker**	**Matrix**	**Abbreviation**	**Method**	**LOQ**
Arachidonic Acid[a]	2,3-dinor-thromboxane B2	Urine	2,3-d-TXB_2	LC-MS/MS	0.05 ng/mL
	Leukotriene E_4	Urine	LTE_4		0.005 ng/mL
	Tetranor-prostaglandin D metabolite	Urine	t-PGDM		0.1 ng/mL
	Tetranor-prostaglandin E metabolite	Urine	t-PGEM		0.1 ng/mL
	2,3-dinor-8-iso prostaglandin F2alpha	Urine	2,3-d-8-iso-PGF2α		0.1 ng/mL
	8-iso prostaglandin F 2alpha	Urine	8-iso-$PGF_{2\alpha}$		0.05 ng/mL
	Prostaglandin F 2alpha	Urine	PGF2α		0.1 ng/mL
	11-dehydrothromboxane B2	Urine	11-dh-TXB_2		0.05 ng/mL
	20-carboxy-leukotriene B4	Urine	20-COOH-LTB_4		0.25 ng/mL
	20-hydroxy-leukotriene B4	Urine	20-OH-LTB_4		0.1 ng/mL

[a] Samples were analyzed at Analytisch-Biologisches Forschungslabor (ABF) GmbH, Semmelweisstr. 5, Planegg, Germany.

LC-MS/MS - liquid chromatography-tandem mass spectrometry; LOQ - limit of quantitation

Scheme S3. Changes in Select Arachidonic Acid Metabolites in Smoking Abstinence.

**Urine Biomarkers**			**Age Cohort**	
**Biomarker (Units)**	**Statistics**	**Time Point**	**24-34 years**	**35-60 years**
LTE_4 (ng/24 hours)	Mean ± SD (*n*)	Day -1	139.1 ± 69.96 (32)	105.5 ± 85.35 (37)
		Day 7	81.04 ± 45.54 (32)	78.1 ± 44.12 (37)
		Day 14	88.9 ± 44.93 (32)	72.4 ± 53.19 (36)
	Percent Change	Day 7 vs. Day -1	-42%*	26%*
		Day 14 vs. Day -1	-36%*	-31%*
2,3-d-TXB_2 (ng/24 hours)	Mean ± SD (*n*)	Day -1	1780 ± 743.57 (32)	1843 ± 946.67 (37)
		Day 7	941.7 ± 433.46 (32)	1331 ± 633.14 (37)
		Day 14	1024 ± 462.26 (32)	1098 ± 617.53 (36)
	Percent Change	Day 7 vs. Day -1	-47%*	-28%*
		Day 14 vs. Day -1	-42%*	-40%*
8-iso $PGF2_{\alpha}$ (ng/24 hours)	Mean ± SD (*n*)	Day -1	566.6 ± 489.96 (32)	434.6 ± 326.96 (37)
		Day 7	365.5 ± 235.87 (32)	413.8 ± 182.52 (37)
		Day 14	403.1 ± 194.66 (32)	414.8 ± 246.73 (36)
	Percent Change	Day 7 vs. Day -1	-36%*	-5%
		Day 14 vs. Day -1	-29%*	-5%

Percent change = (daily mean – baseline mean)/baseline mean x 100, where baseline is Day -1.
* The asterisk represents the statistical significance of the mean difference from baseline ($p<0.05$).

Scheme S4. Bioanalytical Methods for Determining the Biomarkers of Potential Harm – Blood.

**BLOOD**					
**Constituent**	**Biomarker**	**Matrix**	**Abbreviation**	**Method**	**Range***
Complete Blood Count with Differentials and Platelets[a]	White blood cells<br>Red blood cells<br><br>Platelet count<br>Basophils (absolute and [%])<br>Eosinophils (absolute and [%])<br>Lymphocytes (absolute and [%])<br>Monocytes (absolute and [%])<br>Neutrophils (absolute and [%])	Whole blood	WBCs<br>RBCs	Laboratory specific	3.7-11.5 10^9/L<br>FEMALE 3.95-5.21 10^12/L<br>MALE 4.43-5.81 10^12/L<br>155-361 10^9/L<br>0-0.1 10^9/L [0-2%]<br>0-0.5 10^9/L [0.5-7.4%]<br>1.1-3.2 10^9/L [15.8-45.8%]<br>0.3-0.9 10^9/L [4.9-12.8%]<br>1.6-7.7 10^9/L [38.4-74.2%]
Fractional Exhaled Expiratory Nitric Oxide		Breath	FeNO	NIOX Vero®	25-50 parts per billion
Arterial Blood Gases	Bicarbonate<br>Partial pressure oxygen<br>Partial pressure carbon dioxide Oxygen saturation<br>Oxygen Base Excess/Base Deficit<br>pH, Blood	Whole blood	----<br>PAO_2<br>$PACO_2$<br>O_2 Saturation<br>----<br>----	i-STAT System	22-26 mmol/L<br>80-105 mmHg<br>35-45 mmHg<br>95-98%<br>2-3 mmol/L<br>7.35-7.45

[a] Samples were analyzed at Celerion Clinical Laboratory Services, Lincoln, NE.

* Reference ranges stated are for ages 19 years and up (i.e., adults).

Supplementary References

1. Chang, C. M., S. H. Edwards, A. Arab, A. Y. Del Valle-Pinero, L. Yang, and D. K. Hatsukami. "Biomarkers of Tobacco Exposure: Summary of an Fda-Sponsored Public Workshop." *Cancer Epidemiol Biomarkers Prev* 26, no. 3 (2017): 291-302.
2. Luo, X., S. G. Carmella, M. Chen, J. A. Jensen, L. R. Wilkens, L. Le Marchand, D. K. Hatsukami, S. E. Murphy, and S. S. Hecht. "Urinary Cyanoethyl Mercapturic Acid, a Biomarker of the Smoke Toxicant Acrylonitrile, Clearly Distinguishes Smokers from Nonsmokers." *Nicotine Tob Res* 22, no. 10 (2020): 1744-47.
3. Minet, E., F. Cheung, G. Errington, K. Sterz, and G. Scherer. "Urinary Excretion of the Acrylonitrile Metabolite 2-Cyanoethylmercapturic Acid Is Correlated with a Variety of Biomarkers of Tobacco Smoke Exposure and Consumption." *Biomarkers* 16, no. 1 (2011): 89-96.
4. Institute of Medicine Committee to Assess the Science Base for Tobacco Harm Reduction. *Clearing the Smoke: Assessing the Science Base for Tobacco Harm Reduction*. Edited by K. Stratton, P. Shetty, R. Wallace and S. Bondurant, Clearing the Smoke: Assessing the Science Base for Tobacco Harm Reduction. Washington (DC): National Academies Press (US), Copyright 2001 by the National Academy of Sciences. All rights reserved., 2001.
5. McElroy, J. P., S. G. Carmella, A. K. Heskin, M. K. Tang, S. E. Murphy, S. A. Reisinger, J. A. Jensen, D. K. Hatsukami, S. S. Hecht, and P. G. Shields. "Effects of Cessation of Cigarette Smoking on Eicosanoid Biomarkers of Inflammation and Oxidative Damage." *PLoS One* 14, no. 6 (2019): e0218386.
6. Prasad, G. L., B. A. Jones, P. Chen, and E. O. Gregg. "A Cross-Sectional Study of Biomarkers of Exposure and Effect in Smokers and Moist Snuff Consumers." *Clin Chem Lab Med* 54, no. 4 (2016): 633-42.
7. Rångemark, C., G. Ciabattoni, and A. Wennmalm. "Excretion of Thromboxane Metabolites in Healthy Women after Cessation of Smoking." *Arterioscler Thromb* 13, no. 6 (1993): 777-82.
8. Frans, A., N. Gerin-Portier, C. Veriter, and L. Brasseur. "Pulmonary Gas Exchange in Asymptomatic Smokers and Nonsmokers." *Scand J Respir Dis* 56, no. 5 (1975): 233-44.
9. Kullmer, T., H. Kronenberger, R. Siekmeier, and M. Clemens. "[the Value of Studies of Pulmonary Gas Exchange During Exercise for Evaluation of Reduced Arterial Oxygenation at Rest in Asymptomatic Long-Term Smokers]." *Pneumologie* 48, no. 1 (1994): 20-4.
10. Ouattara, S., M. Keita, N. Tuo, C. Dah, E. A. Siransy, and P. Bogui. "[Effect of Smoking on Pao2 at Rest and During Moderate Exercise]." *Dakar Med* 47, no. 1 (2002): 90-5.
11. Kharitonov, S. A., R. A. Robbins, D. Yates, V. Keatings, and P. J. Barnes. "Acute and Chronic Effects of Cigarette Smoking on Exhaled Nitric Oxide." *Am J Respir Crit Care Med* 152, no. 2 (1995): 609-12.
12. Högman, M., T. Holmkvist, R. Wålinder, P. Meriläinen, D. Lúdvíksdóttir, L. Håkansson, and H. Hedenström. "Increased Nitric Oxide Elimination from the Airways after Smoking Cessation." *Clin Sci (Lond)* 103, no. 1 (2002): 15-9.
13. Theophilus, E. H., C. R. Coggins, P. Chen, E. Schmidt, and M. F. Borgerding. "Magnitudes of Biomarker Reductions in Response to Controlled Reductions in Cigarettes Smoked Per Day: A One-Week Clinical Confinement Study." *Regul Toxicol Pharmacol* 71, no. 2 (2015): 225-34.

14. Lei, W., F. Li, X. M. Tang, S. Bian, J. J. Wang, and J. A. Huang. "The Comparision of Two Exhaled Nitric Oxide Analyzers: Niox Vero and Sunvou-Ca2122." *J Breath Res* (2020).
